# Assessment of histology's performance compared with PCR in the diagnosis of *Helicobacter pylori* infection

**DOI:** 10.2144/fsoa-2023-0217

**Published:** 2024-05-14

**Authors:** Manel Moalla, Lassaad Chtourou, Basma Mnif, Slim Charfi, Hend Smaoui, Mon Boudabous, Leila Mnif, Ali Amouri, Hela Gdoura, Adnene Hammami, Tahya Boudawara, Nabil Tahri

**Affiliations:** 1 Gastroenterology department, Hedi Chaker hospital, Sfax, Tunisia; 2 Microbiology department, Habib Bourguiba hospital, Sfax, Tunisia; 3 Pathology department, Habib Bourguiba hospital, Sfax, Tunisia

**Keywords:** diagnostic methods, *Helicobacter pylori*, histology, pathology, PCR

## Abstract

**Aim:** Histology is the most widely used test to detect *H. pylori*. PCR is less used but allows the detection of both infection and antibiotics' resistance. **Methods:** We conducted a monocentric cross-sectional study, collecting 97 symptomatic patients to assess the diagnostic performance of histology in the detection of *H. pylori* infection compared with PCR. **Results:** Sensitivity of histology in comparison with PCR was 81.5% and specificity was 56.3%. A history of anti-*H. pylori* therapy intake, as well as the density of the bacterium on the gastric sample and the presence of gastric atrophy, were significantly correlated to the PCR's result in terms of *H. pylori* detection. **Conclusion:** Thus, histology can be considered as an efficient test compared with PCR in *H. pylori* detection.

## Plain language summary

*Helicobacter pylori* is a type of bacteria that can cause diseases in the stomach and the upper part of the small intestine. A number of different methods are applied by scientists to determine if this bacterium is present. In our research, we specifically examined the accuracy of two types of tests – one where doctors examine tissues under the microscope to find signs of the bacteria (pathological test), and another where they use a method called PCR to find the bacteria's genetic material. Our aim was to determine which test worked better.

## Abstract (plain language summary)

The prevalence of *H. pylori* in symptomatic adults was significant using either histological examination.

Sensitivity and specificity of histology were estimated at 81.5 and 56.3%, respectively, in comparison with PCR.

A history of anti-*H. pylori* treatment, the density of the bacterium on the gastric sample and the presence of gastric atrophy, were significantly related to the PCR result in terms of *H. pylori* detection.

*Helicobacter pylori* (*H. pylori*) infection constitutes a real public health problem. It affects more than 50% of the world's population. Its prevalence varies between 20 and 40% in developed countries and 70% in developing countries [1]. Its implication in the development of several pathologies has been proven. It represents the main cause of gastroduodenal diseases such as peptic ulcer, gastric cancer and mucosa-associated lymphoid tissue (MALT) lymphoma [2,3]. It is involved in some extradigestive pathologies such as iron or vitamin B12 deficiency anemia and idiopathic thrombocytopenic purpura [4].

The diagnosis of *H. pylori* infection is based on invasive or noninvasive methods. Invasive methods include histology, rapid urease test, microbial culture and Polymerase chain reaction (PCR). They all require gastric biopsies [5]. Non invasive methods do not require biopsy and are represented by serology, breath test and stool antigen testing. The prescription of one of these tests depends on the clinical situation. Among the invasive tests, pathological examination of gastric biopsies is the most widely used test in clinical practice. It has proven its high sensitivity and specificity. However, not every curved bacterium is *H. pylori.* Microbial culture has been considered for a long time as the gold standard since its sensitivity and specificity can reach 100%. It requires very specific transportation conditions and needs special replication conditions. Just few laboratories are equipped to isolate this bacterium [6]. Thus, it is proposed usually when antibiotic susceptibility testing is required. On the other hand, PCR which is based on the genomic amplification of *H. pylori* DNA on gastric biopsies, does not require any specific transporting conditions. Many techniques are proposed to improve its sensitivity and specificity such as nested PCR and using very specific primers and internal primers [7,8]. It is admitted now that none of these tests can be considered as gold standard and that the combination of two tests or more increases the diagnostic accuracy [^9-12^].

The aim of our study was to compare the diagnostic performance of pathology in the detection of *H. pylori* infection in comparison with PCR and to investigate the factors associated with positive histology.

## Patients & methods

### Study design & study population

We carried out a cross-sectionnal monocentric study among patients presenting with gastric symptoms referred to the hepato-gastroenterology department of Hedi Chaker Hospital for upper gastrointestinal endoscopy (UE) from March 2017 to February 2020. The patients requiring upper endoscopy with an indication to look for *H. pylori* infection according to Maastricht VI guidelines were included in the study [2]. Patients who refused to participate to the study and those who had received antibiotics in the month before UE were not included in the study. Patients who received anti-*H. pylori* therapy in the last 6 months were excluded from the study.

The endoscopes used were Fujinon^®^ (229A695) or Olympus^®^ (2701213).

Sociodemographic and clinical questionnaire was completed for all patients.

The number of patients to include in the study was calculated based on the sensitivity (95%) and specificity (98%) of histological test and the prevalence of *H. pylori* infection in Tunisia described in the literature (63.7%) [13]. The accuracy of the test was fixed at 10%. The number was estimated at 50 patients at least.

### *H. pylori* detection

For histological analysis, five gastric samples were taken according to Sydney system: two from antrum, two from fundus and one from incisura angularis. The samples were put in a flask containing 10% formaldehyde. *H. pylori* was detected by using hematoxylin-eosin (H&E) stain and modified Giemsa when H&E was negative. Histology was considered positive when it showed active gastritis with individualization of the bacterium.

For PCR, two biopsies were needed: one from antrum and one from fundus. The samples were put in a flask containing saline solution. We used molecular assay using the commercial kit Allplex real-time PCR. DNA was extracted from gastric biopsies using the QIAamp DNA mini kit (QIAGEN, Germany). To detect *H. pylori* infection, an in-house Taqman real-time PCR assay targeting the *H. pylori*-specific gene glmM using forward primer 5′-AGCGCTCTCACTTCCATAGGC-3′, reverse primer 5′-TCTTCGGTTAAAAAAGCGAT-3′ and Taqman probe [5′]6-FAM TGATCCAAATAGGGCCTATGCCTACCCC [3′]3-TAMRA was performed as described in previous studies [14].

### Statistical analysis

Normality of quantitative variables was tested by the Shapiro-Wilk test.

While PCR is not yet the gold standard test for *H. pylori*, we used PCR as a reference to compare the performance (sensitivity and specificity) of PCR to ‘histological examination’. Comparison of qualitative variables was performed by Chi-square test; comparison of continuous quantitative variables was performed by Student's *t*-test.

Sensitivity, specificity, positive predictive value (PPV) and negative predictive value (NPV) of histological test were calculated as well as their 95% CI.

Agreement between the two *H. pylori* diagnostic tests was measured by Cohen's Kappa coefficient. This coefficient is interpreted as follows:

- <0.20: no agreement or very poor agreement;
- 0.21–0.41: poor agreement;
- 0.41–0.60: average agreement;
- 0.61–0.80: good agreement;
- >0.81: very good agreement.

A p-value <0.05 was considered statistically significant. Statistical analysis was carried out using the 20th version of IBM SPSS statistics.

### Ethical consideration

All patients signed an informed consent prior to upper gastrointestinal endoscopy. The study was approved by the Ethics Committee (reference: CPP SOUTH Number 0296/2021).

## Results

### Population study characteristics

We included 124 patients. Twenty-seven patients were excluded since they received an anti-*H. pylori* therapy in the 6 months prior to the study. The final number of the population study was 97. The mean age was 46.8 years ± 16.1. The most well-represented age group was between 50 and 59 years (N = 25; 25.8%). The population study was comprised of 38 men (39.2%) and 59 women with a sex ratio of 0.64. Twenty-six patients (26.8%) were smokers. Regarding digestive history, 13 patients (13.5%) had a personal history of peptic ulcer, 29 (29.9%) had a family history of peptic ulcer and three had a family history of gastric cancer. Twenty patients (20.6%) had arterial hypertension, 13 had diabetes (13.4%) and six had rheumatoid arthritis. Sixteen patients (16.5%) were under proton pump inhibitors.

The indications for upper endoscopy are summarized in Table 1. They were dominated by epigastric pain (N = 71, 73.2%), gastroesophageal reflux disease (GERD) (N = 24, 24.7%), gastrointestinal bleeding (N = 12, 12.4%) and anemia (N = 8, 8.2%).

**Table 1.** Indications of upper endoscopy in our patients.

Indication	Number	Percentage (%)
Epigastric pain	71	73.2
GERD	24	24.7
Digestive bleeding	12	12.4
Anemia	8	8.2
Vomiting	8	8.2
Weight loss	6	6.2
Dysphagia	5	5.2
Diarrhea	3	3.1
Before bariatric surgery	3	3.1

GERD: Gastroesophageal reflux disease.

**Figure 1.**
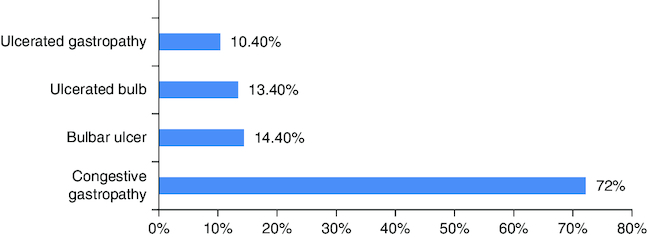
Main endoscopic findings in our patients.

Endoscopic findings were mainly congestive gastropathy (N = 70, 72.2%), bulbar ulcer (N = 14, 14.4%), ulcerated bulb (N = 13, 13.4%) and ulcerated gastropathy (N = 10, 10.4%). These results are illustrated in Figure 1.

Histologically, *H. pylori*-related gastritis was found in 73 patients (75.3%). The density of the bacterium was estimated at very low, mild, moderate and intense in four, 36, 28 and two patients, respectively. It was not mentioned in three patients. Fundal atrophy was noted in 48 patients (49.4%). Fundal intestinal metaplasia was noted in six patients (6.2%).

From the 97 biopsies, 80 (82.5 2%) were positive using PCR.

### Analytic study

From the 97 biopsies, 80 (82.5 2%) were positive for PCR and 73 were positive on histology (75.3%). These results gave a sensitivity of 81.5% (CI = 69.8–86.4) and a specificity of 56.3% (CI = 34.9–76.1) for histology in comparison with PCR. The PPV was estimated at 90.4% (CI = 80.1–94.3). The NPV was estimated at 37.5% (CI = 22.7–56.4).

Chi-square test showed significative association between the two diagnostic tests (p = 3.10^-4^).

Agreement between the two tests measured by Cohen's Kappa coefficient was equal to 0.30. Consequently, agreement between these two tests was poor.

### Factors correlated to the tests' results

No factor had an impact on the histological examination result (Table 2). On the other hand, a history of anti-*H. pylori* therapy intake, as well as the density of the bacterium on the gastric sample and the presence of gastric atrophy, were significantly correlated to the PCR's result in terms of *H. pylori* detection (p = 0.019; p = 0.002 and p = 0.031, respectively).

**Table 2.** Association between anamnestic, clinical, endoscopic and histological data and the pathological test's result.

Data	Studied factor (yes/no)	Histology + (N = 73)	Histology - (N = 24)	p-value
Anamnestic data	Gender (men/women)	29/44	6/15	0.85
	Personal history of peptic ulcer	9/64	4/19	0.53
	Family history of peptic ulcer	22/51	7/17	0.93
	Family history of gastric cancer	3/70	0/24	0.31
	Diabetes	9/64	4/20	0.59
	Arterial hypertension	14/59	6/18	0.54
	Tobacco consumption	19/54	7/17	0.76
	PPI intake	11/62	5/19	0.51
Clinical data	Epigastric pain	54/19	17/7	0.76
	Digestive bleeding	9/64	3/21	0.98
	GERD	17/56	7/17	0.56
	Anemia	4/69	4/20	0.1
Endoscopic findings	Ulcerated gastropathy	8/56	2/22	0.55
	Bulbar ulcer	11/62	3/21	0.56
	Ulcerated bulb	10/63	3/21	0.62
	Congestive gastropathy	52/21	18/6	0.51
Histological findings	Gastric atrophy	36/37	12/12	0.94
	Gastric metaplasia	5/68	1/23	0.54

GERD: Gastroesophageal reflux disease; PPI: Proton pump inhibitor.

## Discussion

In our series, the prevalence of *H. pylori* in symptomatic adults was 75.3 and 82.5% by histological examination and PCR, respectively. This prevalence is consistent with the general prevalence in Africa which is 70% and the prevalence found in Morocco which was 92.6% in asymptomatic people and 89.6% in people suffering from gastric disorders [1,15] and in Tunisia where the prevalence was 87.7% in symptomatic patients in 2005 and 63.7% in 2022 in a multicenter Tunisian study [16]. Our study involved patients who fulfilled the indications for *H. pylori* testing set out in the Maastricht VI consensus [2].

Concerning diagnostic methods, culture was long considered the reference method for diagnosing *H. pylori* infection. Currently, PCR has been shown to be as sensitive as culture in the detection of *H. pylori* and even more sensitive in the evaluation of eradication [17]. As for histology, an Indian review of the literature published in 2014 showed that histology and PCR had a sensitivity and specificity of 66–100%, 94–100% and 75–100%, 84–100%, respectively [8]. The accuracy of histology depends on a number of factors such as the pathologist's experience, the density of *H. pylori* colonization of the gastric mucosa, the quality and quantity of the specimen and the subjective assessment of tissue changes [5]. In addition, the presence of two forms of *H. pylori*: spiral and coccoid could explain the false negatives in histology [18]. As far as PCR is concerned, false positives are due to the detection of Helicobacter non-pylori due to genetic similarity; false negatives due to low bacterial load and presence of PCR inhibitors. However, unlike histology and culture, sample collection, transport and processing do not require any special conditions [5]. Histology compared with PCR requires a more important number of biopsy samples. That may be explained by the patchy colonization of the gastric mucosa by the bacterium. On the other hand, PCR can detect the bacterium DNA even when it is present in very small numbers. Histology needs more time since the gastric samples need to be prepared and stained to be examined which takes usually 2 days or more while PCR provides results in few hours.

PCR is increasingly establishing itself as the reference method for diagnosing *H. pylori*, due to its less stringent requirements and excellent sensitivity and specificity. A French study published in 2020, showed that Allplex™ PCR kit had 100% sensitivity, 97.6% specificity, 98% PPV and 100% PNV in *H. pylori* detection [19]. Indeed, new methods such as nested PCR, which consists in a second amplification on an already amplified sequence, liquid phase (DNA-enzyme immunoassay) and the reverse dot blot line probe assay (LiPA) have demonstrated an increase in sensitivity and specificity in the diagnosis of this bacterium [8].

In our study, sensitivity and specificity of histology were estimated at 81.5 and 56.3%, respectively, in comparison with PCR. The positive and negative predictive values were estimated at 90.4 and 37.5%, respectively. Zsikla *et al.* showed in a Swiss study published in 2006, that PCR can identify *H. pylori* DNA in 20.8% of biopsies with chronic gastritis without histologic detectable bacteria [20]. Compared with the PCR assay considered as gold standard in this study, conventional histology showed a sensitivity of 78.3% and a NPV of 79.2 for the detection of *H. pylori*. This superiority of sensitivity of PCR in gastric biopsy specimens compared with histology and bacterial culture has been described in previous studies [^21-30^]. Yamamura *et al.* explained that by the low density of the bacterium in gastric biopsies because of severe atrophy of the gastric mucosa. Concerning specificity, the studies by Yakoob *et al.* and Mattar *et al.* showed that PCR had higher specificity compared with histology in *H. pylori* detection (98.4 vs 94.2% and 92.3 vs 88.4%, respectively) [27,30]. However, both tests had the same specificity (98%) in a meta-analysis lead by Gisbert *et al.* [28].

In our study, a history of anti-*H. pylori* treatment, the density of the bacterium on the gastric sample and the presence of gastric atrophy, were significantly related to the positivity of PCR result in terms of *H. pylori* detection. These findings meet the results of the studies lead by Yamamura *et al.* and by Hirschl *et al.* in which PCR was found to be more sensitive than histology for detecting *H. pylori* in patients who had received recent antibiotic treatment. PCR detected *H. pylori* DNA in 96% of treated patients, whereas histology only identified bacteria in 52% of cases [21,31].

The strength of our study is that we included a largely sufficient number of patients (N = 97) compared with the number required to test (N = 50). However, this study has some limitations. First, it was only conducted in a single large tertiary university hospital. Second, it concerned only symptomatic patients. Thus, further multicenter studies may be needed to consolidate our findings.

## Conclusion

In our series, the prevalence of *H. pylori* in symptomatic adults was significant using either histological examination (75.3%) or PCR (82.5%). Sensitivity and specificity of histology were estimated at 81.5 and 56.3%, respectively in comparison with PCR. Thus, we can consider histology as an efficient test compared with PCR in *H. pylori* diagnosis. PCR may be more efficient in case of low bacterium load, gastric atrophy and in patients who received anti-*H. pylori* treatment. Given the cost–effectiveness and lack of availability of PCR in our settings, we suggest continuing using histology to detect this bacterium and reserve PCR for antibiotics' resistance detection when necessary.
